# Letting the ‘cat’ out of the bag: pouch young development of the extinct Tasmanian tiger revealed by X-ray computed tomography

**DOI:** 10.1098/rsos.171914

**Published:** 2018-02-21

**Authors:** Axel H. Newton, Frantisek Spoutil, Jan Prochazka, Jay R. Black, Kathryn Medlock, Robert N. Paddle, Marketa Knitlova, Christy A. Hipsley, Andrew J. Pask

**Affiliations:** 1 School of BioSciences, University of Melbourne, Melbourne, Victoria, Australia; 2 School of Earth Sciences, University of Melbourne, Melbourne, Victoria, Australia; 3 Melbourne Museum, Museums Victoria, Melbourne, Victoria, Australia; 4 Czech Centre for Phenogenomics, Institute of Molecular Genetics of the Czech Academy of Sciences, v.v.i., Prague, Vestec, Czech Republic; 5 Tasmanian Museum and Art Gallery, Hobart, Tasmania; 6 School of Psychology, Australian Catholic University, Melbourne, Victoria, Australia; 7 Faculty of Science, Charles University, Prague, Czech Republic

**Keywords:** thylacine, *Thylacinus cynocephalus*, allometry, marsupial, postnatal development, extinct

## Abstract

The Tasmanian tiger or thylacine (*Thylacinus cynocephalus*) was an iconic Australian marsupial predator that was hunted to extinction in the early 1900s. Despite sharing striking similarities with canids, they failed to evolve many of the specialized anatomical features that characterize carnivorous placental mammals. These evolutionary limitations are thought to arise from functional constraints associated with the marsupial mode of reproduction, in which otherwise highly altricial young use their well-developed forelimbs to climb to the pouch and mouth to suckle. Here we present the first three-dimensional digital developmental series of the thylacine throughout its pouch life using X-ray computed tomography on all known ethanol-preserved specimens. Based on detailed skeletal measurements, we refine the species growth curve to improve age estimates for the individuals. Comparison of allometric growth trends in the appendicular skeleton (fore- and hindlimbs) with that of other placental and marsupial mammals revealed that despite their unique adult morphologies, thylacines retained a generalized early marsupial ontogeny. Our approach also revealed mislabelled specimens that possessed large epipubic bones (vestigial in thylacine) and differing vertebral numbers. All of our generated CT models are publicly available, preserving their developmental morphology and providing a novel digital resource for future studies of this unique marsupial.

## 1. Background

**Figure 1.**
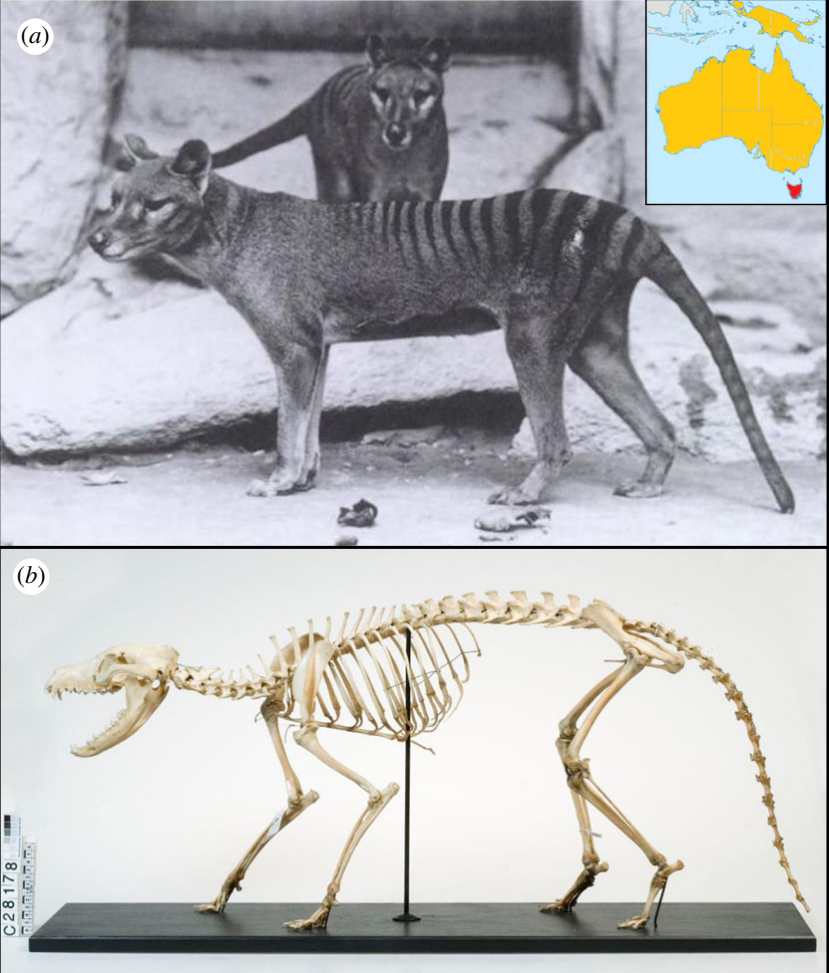
The thylacine. (*a*) Pair of adult thylacines, photograph taken from the US National Zoological Park. The scientific name, *Thylacinus cynocephalus*, translates to ‘dog-headed pouched-dog’, indicating the marsupial's extraordinary resemblance to canids (dogs and wolves), while its striped coat earned it the common name Tasmanian tiger. Inset map shows the historic range throughout Australia and New Guinea (orange), with the final population isolated in Tasmania (red). Map adapted from work by John Tann CC BY 4.0. (*b*) Adult thylacine skeletal morphology (Rodney Start CC BY 4.0).

The thylacine (*Thylacinus cynocephalus*, Harris 1808) was a large Australian marsupial mammal known from the island state of Tasmania, commonly referred to as the Tasmanian tiger or marsupial wolf due to its striped lower back and dog-like appearance (figure 1). Once ranging throughout Australia and New Guinea [1] (figure 1*a*), the thylacine disappeared from the mainland around 3000 years ago, probably through competition and predation by humans [2,3] and dingoes [4,5]. A remnant thylacine population became isolated on Tasmania before they were hunted to extinction in the early twentieth century, with the last known individual dying in captivity in Hobart Zoo in 1936 [6].

### 1.1. The thylacine was a unique marsupial predator

The thylacine, a member of the order Dasyuromorphia, was the largest marsupial carnivore to survive into modern times. Growing up to 35 kg and nearly 2 m in length, the thylacine was three times larger than the Tasmanian devil (*Sarcophilus harrisii*) [7] and possessed unique morphological and behavioural traits associated with a predatory lifestyle. Its overall appearance displayed several independently evolved similarities with placental canids (dogs and wolves), despite the two groups last sharing a common ancestor approximately 160 million years ago [8]. These homologies were especially evident in the thylacine skull, which exhibited numerous convergent adaptions to a carnivorous ecology [9–11], especially when compared to its closest living relative the insectivorous numbat (*Myrmecobius fasciatus*) and other dasyurids, e.g. Tasmanian devil [12]. These include an elongated dog-like snout, long canine teeth and shearing premolars, and a pronounced sagittal crest for muscle attachment [5]. Recent comparisons of three-dimensional cranial shape between the thylacine and other fossil and living mammals showed that this degree of morphological similarity is similar to that found in other textbook examples of phenotypic convergence, making the thylacine-canid comparison an exceptional model of convergent evolution among distantly related taxa [12].

Postcranially, the thylacine also possessed several unique skeletal characteristics distinguishing it from other marsupials. Different numbers of sacral (two instead of the usual three) and caudal (23–25 instead of 20–21) vertebrae separate it from its extant dasyurid relatives [13], and many aspects of its forelimb anatomy appear unlike other meat-eating mammals. For example, the shape of its distal humerus and radius bones indicates supination of the elbow joint and hand—a more generalized mammalian condition that is lost in cursorial pounce and pursuit predators [14,15]. It also experienced an evolutionary reduction of the epipubic bones and clavicles (both present in ancestral mammals), possibly related to its locomotion although this remains speculative [13,16,17]. While these attributes may have assisted in the thylacine's role as a generalist predator, it is unclear to what extent the marsupial mode of reproduction constrained its ability to evolve the more specialized anatomical features as seen in placental carnivorans.

### 1.2. Marsupial reproduction and pouch young development

Marsupials give birth to highly altricial young that undertake an extensive crawl from the urogenital sinus into the mother's pouch where they attach to a teat to continue their development [18]. A fundamental consequence of this reproductive strategy is that certain skeletal elements undergo accelerated development relative to the rest of the body, described as heterochrony. As a result of these early constraints, marsupial neonates exhibit advanced ossification of the forelimbs, shoulder girdle, and facial skeleton at an earlier stage of development than their placental counterparts [19–22]. This is thought to have restricted their evolutionary potential to develop into the diverse range of adult morphologies observed in other mammals [9,23,24]. The retention of a more flexible (supinated) forelimb, as described above, may reflect this developmental constraint [14,15].

The thylacine, like many other quadrupedal terrestrial marsupials, had a backwards-facing abdominal pouch in which it raised litters of up to four young at a time [7]. After one month of gestation in the uterus, the newborn young crawled into the pouch to continue their development while attached to the teat. The pouch young are thought to emerge after a 12 week period, before permanently leaving the pouch after 16 weeks [6,25–27]. While general changes in thylacine skeletal proportions from the juvenile to adult stage have been documented [28], the ontogenetic events that underlie their unique skeletal development during life in the pouch remain largely unknown.

### 1.3. Limited availability of thylacine pouch young specimens

**Figure 2.**
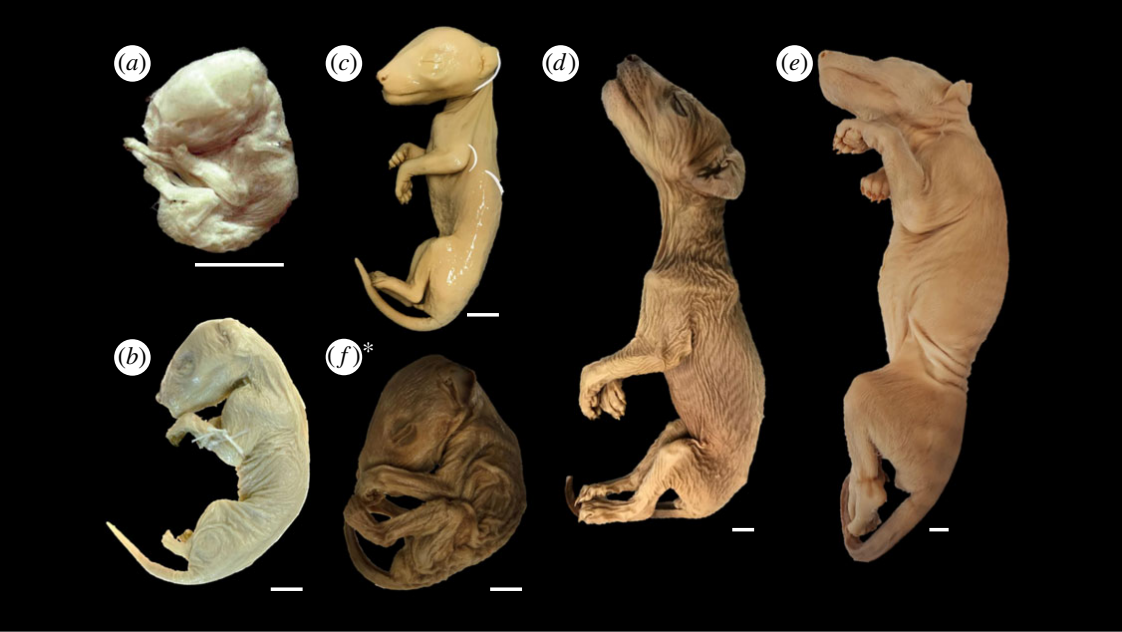
(*a–f*) Thylacine pouch young specimens scanned in this study. (*a*) DZCU8021.1 from a litter of four specimens from Charles University. (*b*) NMV C5755 from a litter of three specimens from Museums Victoria. (*c*) A931, one of two similarly aged specimens, and (*d*) A930, a single individual from the Tasmanian Museum and Art Gallery (TMAG). (*e*) P762, the largest known thylacine pup from the Australia Museum. (*f*) TMAG A934, one of two documented specimens determined to be another species in this study. *Scale bars = 10 mm. *See results §3.3.

The spurious reputation of the thylacine as a sheep killer gained notoriety among early European settlers, leading to its persecution throughout the late nineteenth and early twentieth centuries [6]. Although the predatory nature of the thylacine condemned the animals as vermin, their uniqueness also made them highly sought-after specimens in private and institutional collections, stimulating the trading of whole animals, bones and pelts worldwide [6,27]. Prior to their demise in the early 1900s, over 803 wet and dry specimens were collected and stored in 116 museum and university collections over 23 countries, catalogued in the International Thylacine Specimen Database [27]. In contrast, only 13 registered ethanol-preserved thylacine pouch young are known to date, representing six stages of postnatal development (figure 2) [25,27]. As these individuals are the only preserved pouch young specimens ever likely to be available, they provide a limited yet powerful opportunity to scrutinize the postnatal development of this extraordinary marsupial during a critical phase of its life.

X-ray micro-computed tomography (CT) has become an increasingly popular technique for studying the skeletal anatomy of whole specimens, as it permits visualization of internal features in high resolution without causing damage to the object. In this study, we apply CT to the thylacine pouch young specimens to reconstruct their postnatal ontogeny and generate the first digital developmental series of this extinct marsupial predator. Our high-resolution three-dimensional dataset highlights the skeleton, soft tissues and organs, while providing an overview of anatomy and developmental events that occurred during pouch life. Using precise morphometric measurements of the CT models, we infer the age of each specimen, describe skeletal development and ontogenetic changes, and investigate allometric growth patterns of the thylacine limbs including the identification of limb heterochrony relative to other placental and marsupial mammals. This study provides a developmental framework for identifying the ontogenetic factors underlying the thylacine's unique position among mammals, and together with the recently sequenced thylacine genome [12] offers exciting new opportunities to study the biology of this unique marsupial apex predator.

## 2. Material and methods

### 2.1. Sourcing of material

We used the International Thylacine Specimen Database [27] to locate all known existing thylacine pouch young. Specimens were sourced from Charles University in Prague, Czech Republic (DZCU), and from the following Australian institutes: Museums Victoria (NMV), The Tasmanian Museum and Art Gallery (TMAG), and the Australian Museum in Sydney, New South Wales (AMS) (table 1). As most of the collections contained similarly aged individuals from various litters of young, we gave preference to male individuals for sampling due to the slight sexual dimorphism in marsupials [13]. The exceptions to this were the single Australian Museum specimen, which is female, and the DZCU specimens, where the sex of the young is unknown.

**Table 1.** Specimen identifiers and CT details. List of all known thylacine pouch young with their associated specimen numbers and CT scan parameters. Two specimens marked with an asterisk (*) were not scanned for the present study.

institution	specimen number (s)	crown–rump length (CRL)	resolution/no. projections	voltage (kV)	current (μA)	timing (s)	target, filter
Charles University, Prague	DZCU 8021 (1–4)	26 mm	8.67 µm/1200	90	270	3	tungsten, no filter
Museums Victoria (NMV)	C5755, C5756, C5757	76 mm	17.77–40.38 µm/799	60	650	0.5	tungsten, copper filter
Tasmanian Museum and Art Gallery (TMAG)	A931, A932*	89 mm	49.23 µm/1199	90	440	0.5	molybdenum, aluminium filter
TMAG	A934, A935*	102 mm	49.23 µm/1199	90	440	0.5	molybdenum, aluminium filter
TMAG	A930	167 mm	49.23 µm/1199	90	440	0.5	molybdenum, aluminium filter
Australia Museum (AMS)	P762	214 mm	49.23 µm/1199	90	440	0.5	molybdenum, aluminium filter

### 2.2. Pouch young specimen details

The youngest thylacine specimens were recently discovered in the Zoology Department of Charles University, Prague, labelled as DZCU8021 (table 1 and figure 2*a*), and represents a complete litter of four siblings [25]. Little information is known about the origin of the specimens other than their acquisition into the collection in 1897. The specimens have been previously estimated as ≤2 weeks old based on crown–rump length (CRL) measurements [25].

The next complete litter of young is found in the collection of Museums Victoria, Australia, labelled C5754–C5757 (table 1). The specimens represent a full litter of four pouch young that were received with their mother (C5752) in 1909. All four pouch young were removed from the pouch (and probably the nipples) after their mother was killed in the wild. Of the four young, three remain intact, as the fourth, C5754, was serially sectioned in 1994 and now exists as a series of histological slides. Of the remaining three specimens, C5755 is male (figure 2*b*) and C5756 and C5757 are females. The specimens are estimated to be 4 weeks old based on CRL [25].

The most diverse collection of pouch young exists in Tasmanian Museum and Art Gallery (TMAG), although the ages of the specimens were not known. This collection contains young from three separate litters (table 1): one litter containing one young male and a female sibling (A931 and A932, respectively; figure 2*c*), one litter with two slightly larger pouch young, one with undetermined sex and one male (A933 and A934, respectively; figure 2*f*), and one litter with one well-developed male pup (A930, figure 2*d*). The A930 specimen was brought to TMAG with its female sibling (location currently unknown) and mother as a bounty animal (wild caught) from Campbell Town in 1902.

Finally, a single thylacine pouch young specimen is stored at the Australian Museum in Sydney (table 1). The specimen is a female (P762, figure 2*e*) and is estimated to be approximately 12 weeks old based on X-ray analyses of tooth eruption and CRL [25]. This is the most developed pouch young specimen in any of the collections, and is thought to have started its transition out of the pouch [6]. The specimen was donated to the Australian Museum by the Royal Society of Tasmania in 1866 during an Australian Museum collecting trip led by George Masters. This date of collection makes it the oldest known preserved specimen at over 150 years old.

### 2.3. CT scanning and digital reconstruction

Details of CT parameters are given in table 1. Each specimen was scanned over 360° at varying resolutions using different targets and filters to maximize contrast in the soft tissues. The DZCU specimens were scanned at the Czech Centre for Phenogenomics (Institute of Molecular Genetics ASCR, v.v.i.), Vestec CZ, in a SkyScan 1176 (Bruker microCT, Belgium). The specimens exist as a mounted series of four young suspended in a glass cylinder attached by thread [25] which was placed inside the scanner. All other specimens were scanned at the TrACEES platform, School of Earth Sciences, University of Melbourne, in a phoenix nanotom m (General Electric, USA). Each specimen was wrapped in bubble wrap and mounted in a PVC pipe to minimize movement. The larger individuals (A930 and P762) required 3 and 4 scans, respectively, to capture the full body. These scans were subsequently merged in Avizo (FEI).

Reconstruction of the DZCU specimens was performed in NRecon 1.6.9.15 (Bruker microCT, Belgium) with parameters for smoothing, ring artefact correction and beam hardening correction of 3, 19, and 4%, respectively. The reconstructed three-dimensional volume was exported as a tagged image file format (tiff) stack for further analysis. Automatic noise reduction, thresholding, fluid shape ROI selection, and transposition to three-dimensional matrix were performed with the help of CT analyser 1.16.4.1 (Bruker microCT, Belgium). To ease data operation, original files were downscaled to 25% of their original size in Fiji [29], which also served for converting the data for itk-SNAP 3.6 [30] for three-dimensional segmentation of bony elements. Reconstruction of the Australian specimens was performed in datos|x reconstruction software (GE Sensing & Inspection Technologies GmbH, Wunstorf, Germany). VGStudio Max 3.0 (Volume Graphics GmbH, Germany) and Avizo were used for volume rendering of the three-dimensional CT data, segmentation, and creation of surface meshes. Surface models were re-meshed and simplified in Meshlab (Visual Computing Lab, ISTI, CNR). For organ identification and rendering, subsampled tiff stacks of the specimens were imported into Fiji [29]. Organ layers were visualized in individual slices and highlighted. The altered tiff stacks were reimported to Avizo, which was used for interpolating the segmentation of the layers to a three-dimensional volume. Segmented organs were then visualized by overlaying them in the existing volume using various false colour maps.

CT models of the above specimens are publicly available as stacks of reconstructed tiffs (http://dx.doi.org/10.5061/dryad.5h8k3 [31]), which can be imported into various free and proprietary software for visualization and analysis. In addition, three-dimensional surface models of each specimen can be found in the electronic supplementary materials.

### 2.4. Age estimation

**Figure 3.**
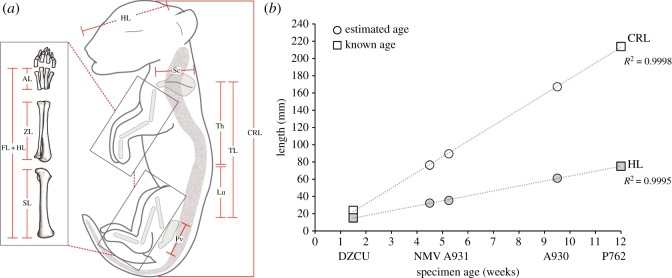
Specimen measurements and age determination of pouch young. (*a*) Linear measurements used in the present study. (*b*) Linear regressions of crown–rump length (CRL) and head length (HL) versus age in weeks for each litter of specimens (taken as the average length) scanned in this study. Ages of the NMV and TMAG (A930, A931) specimens (circles) were calculated and adjusted from previously defined ages of DZCU and AMS (P762) specimens (squares) [25]. Age estimates of the individuals showed a strong linear relationship between age and CRL/HL, as indicated by high *R*^2^ values. Error bars for DZCU and NMV specimens are not visible due to the small size variation between littermates (less than 2.5 mm). Th = thoracic vertebrae, Lu = lumbar vertebrae, TL = trunk length, Sc = scapula, Pv = pelvis, SL = stylopod (humerus/femur), ZL = zeugopod (radius/tibia), AL = autopod (carpals/tarsals), FL+HL = forelimb, hindlimb length.

The approximate ages of the Prague (DZCU), Museums Victoria (NMV) and Australian Museum (AMS) specimens have been previously estimated based on gross morphology and external length measurements [25]; however, the ages of the TMAG specimens remain unknown. We recorded crown–rump length (CRL) and head length (HL) measurements for each of the scanned individuals (*n* = 10) across all litters directly from the reconstructed three-dimensional volumes using the polyline measurement tool in VGStudio Max 3.0 (Volume Graphics GmbH, Germany) to obtain precise measurements to the nearest micrometre (figure 3*a*). Length measurements for multiple individuals of the same litter were averaged and standard error was calculated (figure 3*b*). CRL and HL of the DZCU (*n* = 4) and AMS (*n* = 1) specimens with reported ages [25] were used to calibrate the series. CRL and HL for each of the remaining specimens (*n* = 5) were plotted on the curve. Age estimates were calculated and adjusted based on the best fit of the measurements to the regression line (figure 3*b*).

### 2.5. Allometric scaling

Changes in the proportions of body parts during growth, known as allometry, are common in mammals and are thought to underlie morphological diversification within and among species [32–35]. The highly altricial birth of marsupial neonates, in which only the musculoskeletal systems of the anterior postcranium (forelimbs, shoulder girdle) and oral apparatus are well developed, implies that the majority of allometric changes occur inside the pouch and are potentially limited by functional constraints in early ontogeny (see references in [36]). However, how these regions scale with each other and with body size in general is largely unknown in carnivorous marsupials, which may experience different selection pressures related to feeding and locomotion than do placentals and other non-carnivorous marsupials. We therefore examined allometric growth patterns of the thylacine pouch young during ontogeny, and compared them to the observed developmental trajectory in juveniles through to adulthood [28].

We recorded skull (condylobasal length), forelimb (sum of the length of the humerus, radius and third metacarpus), scapula (anterior to posterior point), hindlimb (sum of the length of the femur, tibia and third metatarsus), pelvis (anterior to posterior point), and trunk length (sum of the length of the thoracic and lumbar vertebrae) (figure 3*a*) to match previously published juvenile and adult growth data [28]. Body proportions were calculated as the length of each component divided by trunk length, given as a percentage of specimen's total trunk length (% TL), and compared across all developmental time points.

We also examined allometric scaling of the limb long bones throughout the entire ontogeny of the thylacine using methods described by Kilbourne & Makovicky [33]. We recorded length (mean of left and right bone) and circumference (the mean of the anterior–posterior and medial–lateral width of the midshaft *π) of the long bones of the forelimb (humerus and radius) and hindlimb (femur and tibia). Pouch young long bone measurements were taken from the digital reconstructions using the poly-line tool in VGStudio Max 3.0 (Volume Graphics GmbH, Germany) and similar tools in CT analyser 1.16.4.1 (Bruker microCT, Belgium) and tpsDig2 [37]. Juvenile and adult long bones were measured from specimens within the collection at Museums Victoria using digital callipers and a flexible tape measure.

To test for allometric scaling, length and mid-shaft circumference of each long bone were natural log-transformed and used to generate reduced major axis (RMA) regression slopes. Natural log-transformed data were input into RMA v. 1.21 [38] and slopes with 95% confidence intervals (CIs) were generated using 10 000 bootstraps. Cases in which both the slope and CI were greater than 1.0 were considered as positive allometry (growing more slender), 1.0 as isometry (maintaining a constant proportion), and below 1.0 as negative allometry (growing more robust) [33]. Allometric scaling of thylacine long bones was compared with other published datasets containing similar measurements of placental and marsupial mammals.

## 3. Results and discussion

### 3.1. Developmental staging of specimens

To determine the ages of the unknown specimens, we reclassified the complete series of known pouch young using CRL and HL measurements to establish a growth trajectory. CRL is used as the most accurate means of ageing developing marsupial pouch young, as it has been shown to be linear with age in dunnarts [39], Tasmanian devils [40], quolls [41], bettongs [42] and tammar wallabies [43]. We calibrated the growth series using the previously estimated ages of the youngest DZCU specimens (≤2 weeks) and oldest AMS specimen (12 weeks) [25] (figure 3*b*, squares). TMAG specimen A934 was omitted from the series due to its unusual morphology (see §3.3). To fit the regression line we interpolated and adjusted the ≤2 week age estimate for the DZCU specimens to 1.5 weeks, and retained the AMS specimen's estimate of 12 weeks based on X-ray analysis of skull and dentition [25]. We then adjusted the CRL and HL measurements of the specimens with unknown ages (figure 3*b*, circles) to the regression line until the data showed a strong linear fit (*R*^2^ > 0.999). Our newly established growth curve resulted in age estimates for the NMV young of approximately 4.5 weeks old, half a week older than previously described [25], 5.25 weeks for TMAG specimen A931, and 9.5 weeks for TMAG A930 (figure 3*b*). Despite our small sample sizes, these ages fall within a half a week of those previously estimated, again supporting the strong linear relationship between CRL and age in marsupials.

### 3.2. Descriptions of thylacine pouch young

#### 3.2.1. DZCU8021: 1.5 weeks old

**Figure 4.**
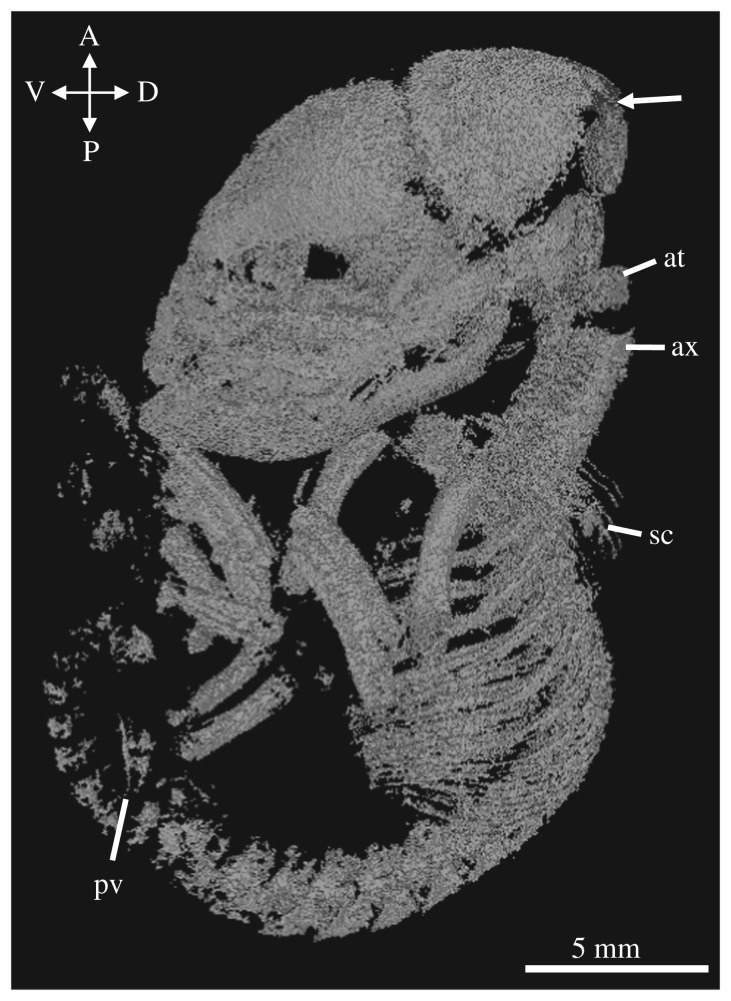
Skeletal reconstruction of DZCU 8021. Lateral view of the reconstructed 1.5-week-old thylacine pouch young. Arrow indicates open sutures of the neurocranium. at = atlas, ax = axis, sc = scapula, pv = pelvis.

The DZCU specimens represent the earliest stage of thylacine postnatal development and appear of a generalized marsupial neonate morphology (figures 2*a* and 4 and electronic supplementary material, figure S1). They display characteristic precocial development of the forelimbs and highly altricial hindlimbs, showing similarities with other equally staged dasyurid marsupials [39,44], American opossums [20], Australian brushtail possums and bandicoots [19], demonstrating that the thylacine shared a similar early marsupial ontogeny and was probably subjected to the same musculoskeletal constraints associated with the crawl to the pouch [23,45]. At this early stage of development the young would have been pulled off the teat of their mother, which may have resulted in some disfiguration of the specimens [25]. The overall morphology of the specimens suggests they were originally compacted in a small vessel before being mounted on thread in their current configurations. We were able to identify bony skeletal elements, though soft tissue detail was limited.

The 1.5-week-old thylacine pouch young had already undergone a significant amount of osteogenesis of the cranial and postcranial skeleton. The skull was large compared to the body (74% TL) and all the major bones were visible. The facial prominence and mandibles show an elevated level of ossification and suture closure compared with the rest of the cranium, probably owing to the constraints of suckling of the altricial neonate [36]. Two to three tooth sockets are visible in each of the jaw quadrants. Many of the sutures between the major bones remain open, especially between the frontal, parietal, squamosal and occipital bones surrounding the neurocranium.

Ossification of the axial skeleton was present with clear segmentation of the vertebrae, ribs and early ossification centres of the sternum. The atlas and axis were large, probably aiding in support of the cranium during the crawl to the pouch and suckling. The young had three sacral vertebrae, characteristic of the thylacine [13]. The caudal vertebrae were difficult to identify due to the low degree of ossification at this stage of development. The limbs displayed clear heterochrony in their state of development, recapitulating the known early development in marsupials, with the forelimbs being longer and larger than the hindlimbs. The young showed accelerated development of the long bones of the forelimbs (58% TL) and shoulder girdle, compared with the bones of the hindlimb (40% TL), assisting in the immediate post-birth crawl to the pouch [18]. The bones of the hindlimb and two small ossification centres in the developing pelvis could be seen, though the specific pelvic bones were difficult to identify. The young displayed early ossification of the metacarpals, metatarsals and digits, but no obvious bones of the carpus or tarsus.

#### 3.2.2. TMAG A931: 5 weeks old

**Figure 5.**
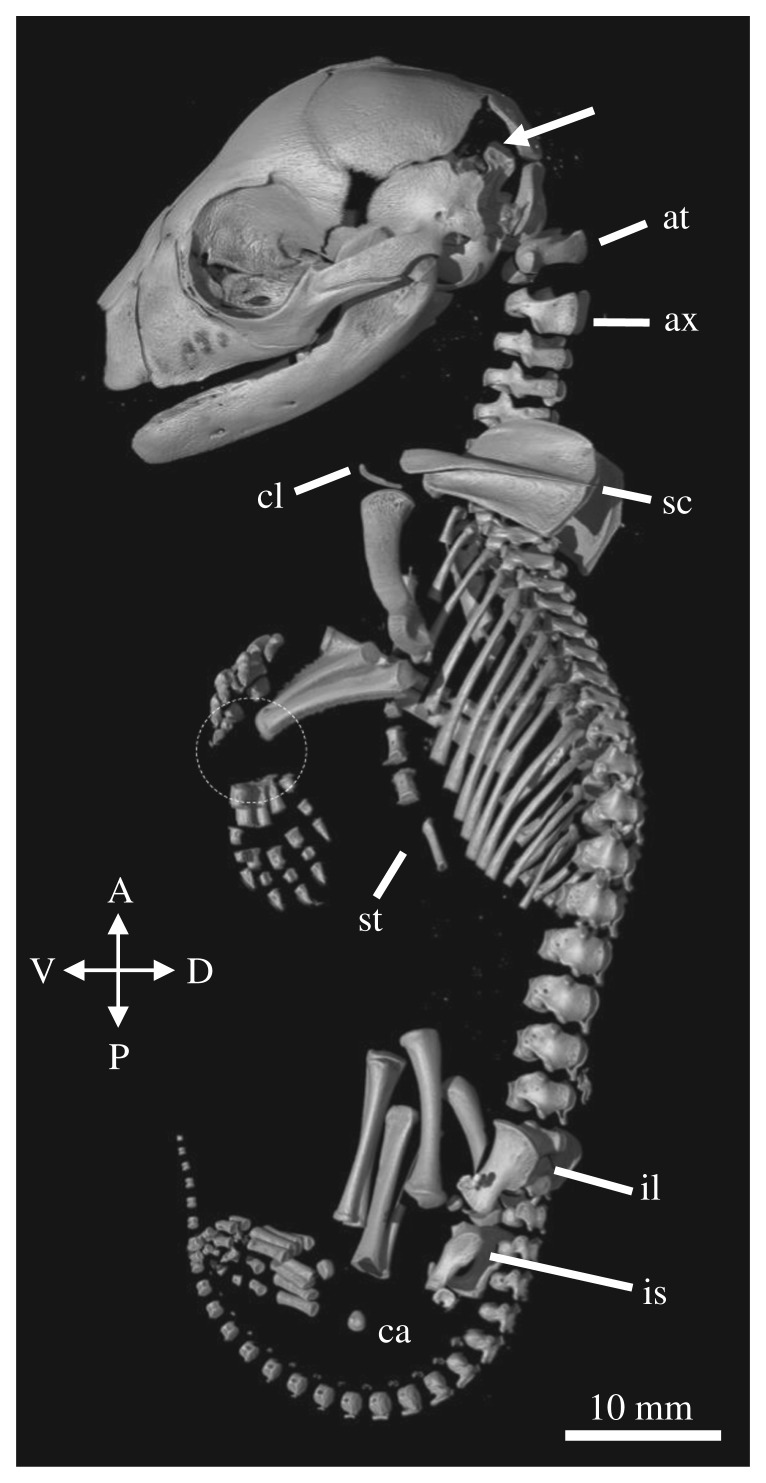
Skeletal reconstruction of TMAG A931. Lateral view of the reconstructed 5.25-week-old thylacine pouch young. Arrow indicates open sutures of the neurocranium. Dashed circle shows the lack of ossified carpal elements. at = atlas, ax = axis, ca = calcaneus, cl = clavicles, il = ilium, is = ischium, sc = scapula, st = sternum.

The TMAG A931 pouch young was similar in size and state of development to the NMV specimens (figure 2*b*,c and electronic supplementary material, figure S3); however, since the former appeared in a better state of preservation, we limit our description to the single specimen. By 4.5–5.25 weeks the thylacine pouch young presented a less generalized morphology and shared similarities with other young dasyurid marsupials [39,44]. TMAG A931 displayed some distinct surface features, such as delamination of the oral fissures, but lacked characteristic dorsal striping. The young was virtually hairless with some vibrissae appearing on the face and head, consistent with previous observations [46]. CT reconstructions of both sets of young showed similar skeletal morphologies, though the TMAG specimen showed higher resolution of its soft tissues compared with the NMV young. We were able to clearly identify the heart, lungs and liver in the reconstructions; however, the brain was harder to visualize (data not shown) (figures 2*c* and 5 and electronic supplementary material, figure S2).

At 4.5–5.25 weeks of age the thylacine possessed a largely ossified skeleton. All bones of the facial skeleton were present, albeit short, with near-complete closure of sutures and development of several deciduous teeth embedded in the upper and lower jaws. The sutures of the neurocranium had begun to close, especially between the frontal and parietal (though not complete), but remained open between the frontal, parietal and squamosal, and between the parietal, squamosal and occipital bones. Despite the advanced level of ossification of the skull, the young still displayed a generalized neonatal cranial shape seen in other marsupials [47], and disparate to its adult morphology.

The postcranial skeleton had undertaken a substantial increase in its relative size compared with the cranium (59% TL), and had undergone ossification of all the major bones. The various vertebrae segments were all present, including the enlarged axis and atlas, though medially unfused and disconnected from one another. The young possessed two unfused sacral vertebrae and 24 detectable caudal vertebrae, confirming it was a thylacine [13]. The pouch young displayed an ossified sternum and the small vestigial clavicles were apparent. The shoulder girdle (scapula) was large and pronounced, the forelimb long bones had elongated and there was ossification of the digits. Notably, the forelimb was lacking any ossification of the carpals in the wrist. The hindlimb long bones had elongated (56% TL), though they were still shorter than the forelimbs (61% TL). The digits were present and the first bone of the tarsus (the calcaneus) had ossified. Both limbs showed development of the claws. The pelvic girdle contained unfused ilium and ischium bones and two small ossification centres for the bones of the pubis. The marsupial-specific epipubic bones were absent, a characteristic feature of the thylacine [13,48].

#### 3.2.3. TMAG A930: 9.5 weeks old

**Figure 6.**
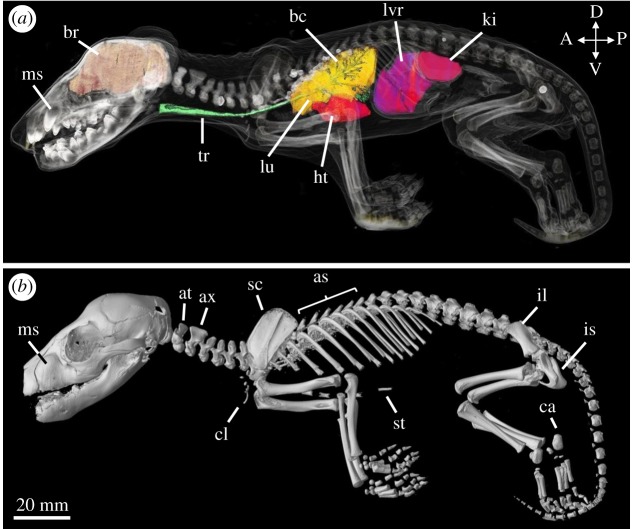
Soft tissue and skeletal reconstructions of TMAG A930. (*a*) Lateral view of the 9.5-week-old thylacine with semi-transparent skin, internal organs and bony elements. The brain (orange), trachea (green), lungs (yellow), heart (red) liver (purple) and kidneys (pink) were separately rendered and overlaid. Teeth showed the highest density (white) followed by the bones of the cranium and limb bones. Lighter elements, including the skin, appear translucent. (*b*) Lateral view of the reconstructed pouch young. as = axial spines, at = atlas, ax = axis, bc = bronchioles, br = brain, ca = calcaneus, cl = clavicles, ht = heart, il = ilium, is = ischium, ki = kidney, lu = lung, lvr = liver, ms = maxillary swellings, sc = scapula, st = sternum, tr = trachea.

By 9.5 weeks, the pouch young possessed a more recognizable thylacine morphology with a covering of fur (though the stripes were not yet visible), numerous facial vibrissae, and sharp claws and fleshy footpads on both sets of limbs (figures 2*d* and 6*a*,*b* and electronic supplementary material, figure S4). The exceptional state of preservation of the young was evident in the CT reconstructions, displaying clear contrast of both skeletal elements and soft tissues, allowing rendering of many of the key internal organ systems, specifically the brain, heart, lungs, liver and kidneys.

The cranial morphology of the young was at a substantially advanced stage, displaying a highly ossified facial skeleton and elongation of the facial bones. The maxillary bones contained two large swellings on either side of the upper jaw housing the pre-erupted canines, and several other dense, pre-erupted teeth were housed in the upper and lower jaw. The sutures of the neurocranium were almost closed, with the remaining open sutures towards the ventral edge of the parietal and occipital bones.

The postcranial morphology of the young displayed many key differences compared with early developmental stages. Despite the elongation of the skull, the head was 53% the length of the trunk, suggesting a rapid expansion of other bony elements. The axial skeleton was well developed with the initial interlocking of the vertebrae and closure of the medial sutures, creating the axial spines in the thoracic vertebrae. The pelvic region contained two sacral vertebrae, and 24 caudal vertebrae in the tail confirming it was a thylacine. The vestigial clavicles were reduced in relation to the surrounding bones of the pectoral girdle, and no visible epipubic bones were present in the pelvic region. The limbs had undergone a heterochronic shift in their size and state of development, with the hindlimbs (68% TL) overtaking the length of the forelimbs (65% TL), and many of the bony elements of the carpals and tarsals had ossified.

#### 3.2.4. AMS P762: 12 weeks old

**Figure 7.**
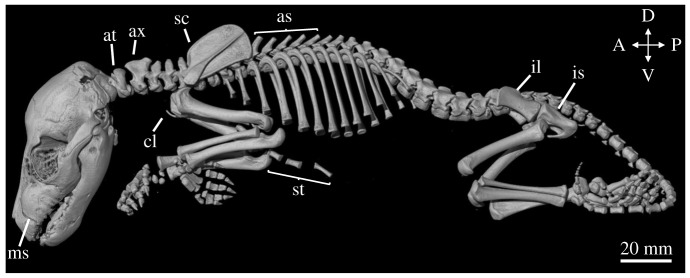
Skeletal reconstruction of AMS P762. Lateral view of the reconstructed 12-week-old thylacine pouch young. as = axial spines, at = atlas, ax = axis, ca = calcaneus, cl = clavicles, il = ilium, is = ischium, ms = maxillary swellings, sc = scapula, st = sternum.

By 12 weeks, the thylacine pouch young displayed a distinct juvenile morphology with a marked increase in size (figures 2*e* and 7 and electronic supplementary material, figure S5). Several external features were present and have been previously discussed in detail by Boardman [46], including distinct facial features, fur covering, and the presence of a pouch. The young had developed several of its characteristic stripes down its lower back and tail, a feature absent in the earlier pouch young specimens. The excellent preservation of the young, similar to the 9.5-week-old specimen TMAG A930 (figure 2*d*), allowed three-dimensional reconstructions of high resolution and clarity. The soft tissue and internal organ systems were clearly visible in the young, including the brain, heart and lungs, though as the organs from the abdominal cavity had previously been removed we did not render the organs.

The skeleton of the 12-week-old pouch young was well developed and highly ossified. The overall morphology of the pouch young was similar in appearance to the 9.5-week-old specimen (TMAG A930; figure 6) though showed an increase in its state of development and size. The bones of the cranium were all present and most of the sutures were closed, with the only remaining open sutures surrounding the posterior bones of the skull. The facial prominence had elongated, overall increasing the length of the skull (55% TL), the canines remained within the maxillary swellings, and several small teeth were present in the upper and lower jaws (not shown).

The postcranial skeleton had fused, interlocking vertebrae creating several axial spines down the specimen's back. The clavicles were small and reduced and the epipubic bones were absent. The limbs had increased in their overall length in relation to the trunk, and the hindlimbs (80% TL) were longer than the forelimbs (74% TL). Most of the smaller bones of the carpus and tarsus were ossified and the metatarsals and digits were elongated in the hindlimb compared with the metacarpals and digits of the forelimb. The fortification of the skeletal elements were probably due to the musculoskeletal requirements of imminent exit from the pouch, and the gain of semi-independence.

### 3.3. Misidentified specimen TMAG A934

**Figure 8.**
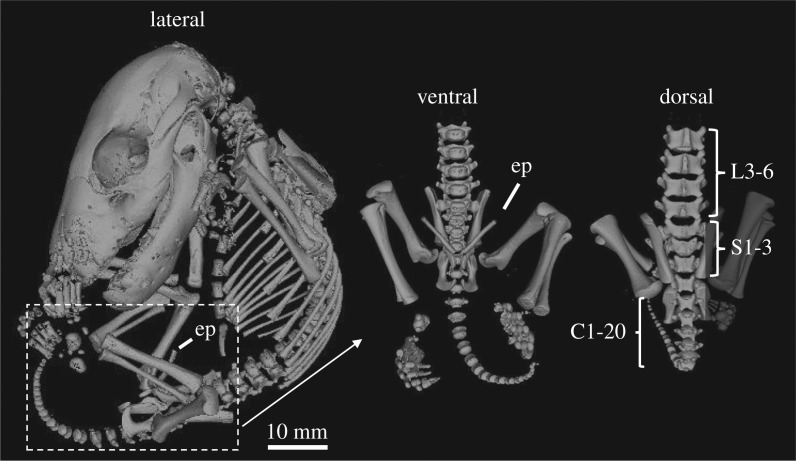
Skeletal reconstruction of TMAG A934. Lateral view of the reconstructed specimens (left). Interestingly, the reconstructions displayed several anomalies compared with the other pouch young specimens, including two large ossified epipubic bones (ep), and three sacral and 20 caudal vertebrae (right), both features known to be absent in the thylacine.

External observations of the TMAG A934 specimen showed a similar morphology to the other thylacine pouch young, and displayed a fine dark fur over its body with numerous vibrissae on the head (figures 2*f* and 8 and electronic supplementary material, figure S6). However, skeletal reconstructions revealed several key differences compared with the other thylacine specimens. Within the pelvic girdle was the presence of two large epipubic bones, a marsupial-specific feature that has become vestigial in the thylacine, remaining only as two small cartilaginous protrusions [48]. The specimen also possessed three sacral vertebrae and 20 detectable caudal vertebrae, a characteristic feature of other dasyurid species [13]. These distinctive features suggest that the pouch young were incorrectly labelled as thylacine and instead are more likely a quoll (*Dasyurus* sp.) or Tasmanian devil (*Sarcophilus harrisii*). Based on these observations, this specimen was excluded from our analyses and is pending genetic sequencing for species identification.

### 3.4. Allometric growth patterns during thylacine ontogeny

**Figure 9.**
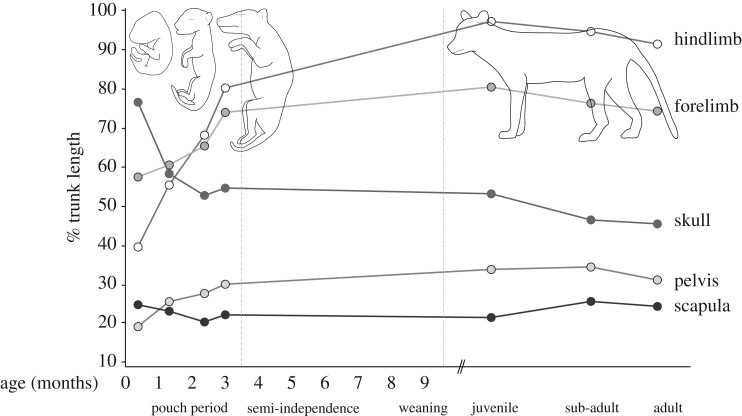
Growth-dependent changes in the skeletal proportions of the thylacine. Changes in the ratios of skeletal components throughout the entire developmental tragectory of the thylacine, as a proportion of total trunk length. Juvenile, sub-adult and adult measurements taken from Moeller [28].

Using measurements of the thylacine pouch young, we explored allometric changes in the skull and appendicular skeleton throughout its entire ontogeny. At 1.5 weeks old, the DZCU specimens represent the earliest documented stage of postnatal thylacine development, and display acceleration of the anterior skeleton (skull, scapula and forelimb bones) compared with the posterior elements (hindlimbs and pelvis) (figure 9). The skull was relatively large in the neonate (76% TL), probably in aid of support during suckling, before rapidly reducing in its overall size to roughly half its total trunk length in the following weeks of development (figure 9).

**Figure 10.**
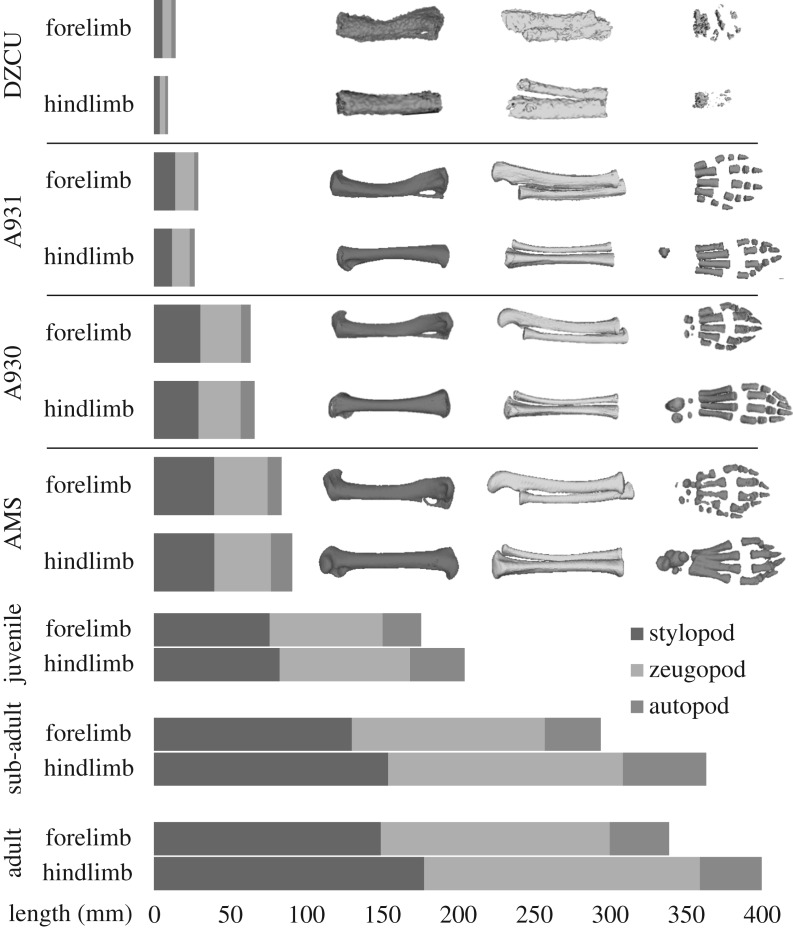
Limb heterochrony in the thylacine. Relative lengths of thylacine forelimb and hindlimb elements throughout development, shown as shaded grey bars. The thylacine forelimbs begin larger and longer than the hindlimbs, before being overtaken within the first half of pouch development. Limbs are divided into proximal and distal segments: the stylopod (humerus and femur), zeugopod (ulna/radius and tibia/fibula) and autopod (carpals, metacarpals, tarsals metatarsals and digits).

Heterochrony of the thylacine limbs was also distinct in the early neonate (forelimb 58% TL versus hindlimb 40% TL), including a larger scapula compared with the pelvis (26% TL and 20% TL, respectively). After 5 weeks the forelimb was still slightly larger than the hindlimb (60% TL versus 55% TL), although the scapula was now proportionally smaller than the pelvis (24% TL and 26% TL, respectively) and maintained this state throughout ontogeny. The developmental lag of the hindlimb was overcome after approximately 8 weeks of pouch development, with the hindlimbs distinctly longer than the forelimbs by 9.5 weeks (68% TL versus 65% TL) (figure 9). In the 2.5 weeks prior to emergence from the pouch, the limbs (especially hindlimbs) had grown rapidly in length (forelimb 74% TL, hindlimb 80% TL), presumably to support the weight and independent locomotion of the pup. The hindlimbs continued to grow longer than the forelimbs throughout the juvenile and adult stages (figures 9 and 10). Therefore, despite its unique adult morphology, the thylacine was subject to the same conserved ontogenetic growth trajectories to that observed in other marsupials [32,49–51].

Individual bones of the thylacine limbs also displayed marked differences in allometric scaling during growth (figure 10). Following birth, the stylopodial (humerus, femur) and zeugopodial (radius, tibia) elements of the fore- and hindlimbs were roughly equal in length, but the autopod of the forelimb (carpus) was longer than that of the hindlimb (tarsus). These proportions were maintained until approximately 5.25 weeks, when the length of the tarsus overtook that of the carpus. By 9.5 weeks the stylopodia were longer than the zeugopodia, persisting until emergence from the pouch at 12 weeks. During the transition of semi-independence (returning to suckle) to an independent juvenile, the length of the zeugopodial elements of the hindlimb surpassed that of the stylopodia. These limb bone ratios were maintained through to adulthood with the hindlimbs ultimately reaching approximately 1.2 times the length of the forelimbs [52].

Despite observed differences in development of the fore- and hindlimbs, growth of the long bones in both sets of limbs scaled with positive allometry from 1.5 weeks through to adulthood (table 2). For all measured elements (electronic supplementary material, table S1), slopes ranged from 1.2 to 1.4 (95% CI 1.15, 1.51). Scaling of length with circumference was similar for all bones except the radius, which had a steeper slope meaning that as it grew longer, it became proportionately more slender. For all long bones *R*^2^ was close to 1 (>0.99), indicating a tight relationship between these variables throughout ontogeny (table 2; electronic supplementary material, figure S7). While the early pouch young had yet to develop ossified long bone epiphyses, the exclusion of these specimens from the analyses yielded similar overall positive allometric growth trends (data not shown).

**Table 2.** Ontogenetic allometry of thylacine long bones. Reduced major axis (RMA) analysis of allometric growth patterns of thylacine forelimb (humerus and radius) and hindlimb (femur and tibia) bones with comparisons to published marsupial [32,53] and placental [33] data. All four thylacine long bones scaled with positive allometry throughout ontogeny, similar to that seen in placental carnivores (i.e. *Panthera tigris*, *Canis latrans*), but dissimiar to growth rates observed in other marsupials and placental herbivores *Cetartiodactyla*. + positive allometry; − negative allometry; iso, isometry.

	humerus						radius						
taxon	*n*=	Y-int	slope	95% CI limits	*R*^2^	allometry	*n*=	Y-int	slope	95% CI limits	*R*^2^	allometry	ref
marsupials													
***Thylacinus cynocephalus (Tasmanian tiger)***	13	0.648	1.198	1.154, 1.242	0.997	+	13	0.757	1.403	1.321, 1.486	0.992	+	this study
*Trichosurus Vulpecula* (*brushtail possum*)^a^	51	nd	0.887	nd	0.950	−	51	nd	0.780	nd	0.931	−	[32]
*Macropus eugenii* (*tammar wallaby*)^a^	25	nd	0.917	nd	0.957	−	24	nd	0.620	nd	0.945	−	[32]
*Monodelphis domestica* (*short-tailed opossum*)^a^		nd	0.710	nd	nd	−		nd	0.850	nd	nd	iso	[53]
placentals													
*Carnivora*													
*Canis latrans* (*coyote*)	13	−1.183	1.720	1.601, 1.884	0.974	+		nd	nd	nd	nd		[33]
*Panthera tigris* (*tiger*)	13	−0.517	1.360	1.283, 1.473	0.983	+	12	−0.002	1.330	1.245, 1.440	0.986	+	[33]
*Ursus americanus* (*black bear*)	19	0.095	1.240	0.974, 1.347	0.966	iso	16	1.372	1.370	0.843, 1.161	0.939	iso	[33]
*Cetartiodactyla*													
*Connochaetes taurinus* (*blue wildebeest*)	15	1.714	0.800	0.738, 0.875	0.976	−	13	1.844	0.860	0.805, 0.924	0.988	−	[33]
*Bison bison* (*bison*)	16	1.785	0.790	0.707, 0.908	0.958	−	12	1.560	0.870	0.805, 0.995	0.971	−	[33]
*Proboscidea*													
*Loxodonta africana* (*African elephant*)	11	1.632	0.900	0.618, 1.038	0.946	iso	7	1.849	0.860	0.632, 0.946	0.984	iso	[33]
	femur						tibia						

taxon	*n*=	Y-int	slope	95% CI limits	*R*^2^	allometry	*n*=	Y-int	slope	95% CI limits	*R*^2^	allometry	ref
marsupials													
***Thylacinus cynocephalus (Tasmanian tiger)***	13	0.660	1.241	1.179, 1.303	0.994	+	13	0.690	1.277	1.211, 1.344	0.994	+	this study
*Trichosurus Vulpecula* (*brushtail possum*)^a^	51	nd	0.922	nd	0.867	+	51	nd	0.876	nd	0.944	−	[32]
*Macropus eugenii* (*tammar wallaby*)^a^	25	nd	0.854	nd	0.965	−	22	nd	0.402	nd	0.716	−	[32]
*Monodelphis domestica* (*short-tailed opossum*)^a^		nd	0.890	nd	nd	iso		nd	0.890	nd	nd	iso	[53]
placentals													
*Carnivora*													
*Canis latrans* (*coyote*)	13	−0.788	1.640	1.457, 2.087	0.949	+	9	−1.883	1.98	0.567, 2.263	0.905	iso	[33]
*Panthera tigris* (*tiger*)	15	−0.721	1.460	1.389, 1.581	0.985	+	12	−0.736	1.45	1.319, 1.651	0.98	+	[33]
*Ursus americanus* (*black bear*)	19	0.266	1.270	0.965, 1.395	0.959	iso	16	1.091	1.04	0.863, 1.138	0.95	iso	[33]
*Cetartiodactyla*													
*Connochaetes taurinus* (*blue wildebeest*)	15	1.261	0.960	0.920, 1.017	0.989	−	15	1.851	0.88	0.8268, 0.9357	0.989	−	[33]
*Bison bison* (*bison*)	16	1.427	0.920	0.8634, 0.9969	0.975	−	17	2.232	0.75	0.6370, 0.8947	0.932	iso	[33]
* Proboscidea*													
*Loxodonta africana* (*African elephant*)	11	1.565	0.950	0.700, 1.069	0.972	iso	10	1.201	0.97	0.597, 1.096	0.94	iso	[33]

^a^RMA calculated with long bone midshaft width.

Large-bodied (greater than 20 kg) mammals are generally associated with isometric or negative growth rates, as increased body mass imposes greater mechanical stress on the long bones during ontogeny (e.g. black bear and elephant (isometric), wildebeest and bison (negative) [33]; table 2). In contrast, limb bones of large placental carnivorans such as canids and felids tend to scale with positive allometry [33]. This divergence in allometric scaling is probably facilitated by locomotor specializations such as limb posturing, muscle mechanics, and behaviour, that mitigate mass-specific forces on the skeleton [54,55]. Although the adult thylacine had relatively short legs for its large body size [15], we found patterns of positive allometric scaling consistent with canids and felids (e.g. coyote, tiger; table 2), suggesting it may have evolved similar adaptive strategies to overcome size-related constraints [33,54].

Locomotor behaviour of the thylacine is still under debate [14,15,56]; however, our results support reconstructions of the animal as a cursorial predator despite its constrained marsupial ontogeny. Although comparable studies on marsupial allometry are limited, measurements of long bone length versus midshaft diameter in smaller taxa like tammar wallaby (*Macropus eugenii*), brushtail possum (*Trichosurus vulpecula*) and short-tailed opossum (*Monodelphis domestica*) reveal isometric or negative allometric growth rates (table 2) [32,53]. Further sampling of other larger bodied and predatory marsupials may help to distinguish the relative influences of ecology and phylogeny on thylacine development, determine its uniqueness among marsupials and uncover the developmental processes leading to extraordinary phenotypic convergence between mammals.

## 4. Conclusion

The limited availability of thylacine pouch young specimens and the lack of non-invasive techniques to interact with them has so far restricted studies of its ontogenetic development. Using key morphometric parameters based on CT data, our growth series has refined the staging of these specimens into five unique postnatal time points covering approximately 1.5, 4.5, 5.25, 9.5 and 12 weeks of age. As the thylacine started its journey from the pouch after 12 weeks [6], this growth series represents the entire critical window of development in the pouch. Furthermore, our study also revealed the incorrect classification of one thylacine specimen (TMAG A934) and its littermate, reducing the number of known intact thylacine pouch young to 11 individuals worldwide. This finding clearly demonstrates the power of CT technology for taxonomic identification of rare specimens.

Using comparative measurements of skeletal elements, we illustrate how the thylacine matured from a generalized marsupial neonate into an adult with allometric patterns resembling a cursorial placental carnivore. Although our analyses mostly focused on morphology of the postcranial skeleton, our future work will describe cranial ontogeny of the thylacine pouch young specimens and its role in adult convergence with placental canids.

This publicly available series, together with the recent publication of the thylacine genome [12], provides a linked genetic and morphological dataset allowing further investigations into the development of this unique species. The approaches used here, taking advantage of recently developed techniques in X-ray computed tomography and three-dimensional visualization, have allowed us to preserve the virtual morphology of this extinct animal and provide a valuable resource for future studies.

## Supplementary Material

Supplementary figure 1

## Supplementary Material

Supplementary figure 2

## Supplementary Material

Supplementary figure 3

## Supplementary Material

Supplementary figure 4

## Supplementary Material

Supplementary figure 5

## Supplementary Material

Supplementary figure 6

## Supplementary Material

Supplementary figure 7

## Supplementary Material

Supplementary table 1

## Data accessibility

All reconstructed specimen tiff image stacks have been uploaded to the online depository Dryad and are publicly accessible (http://dx.doi.org/10.5061/dryad.5h8k3) [31].
