# Supplementary figures and images for: Letting the ‘cat’ out of the bag: pouch young development of the extinct Tasmanian tiger revealed by X-ray computed tomography
Source: R Soc Open Sci. 2018 Feb 21;5(2):171914. doi: 10.1098/rsos.171914 (PMC5830782; doi:10.1098/rsos.171914)

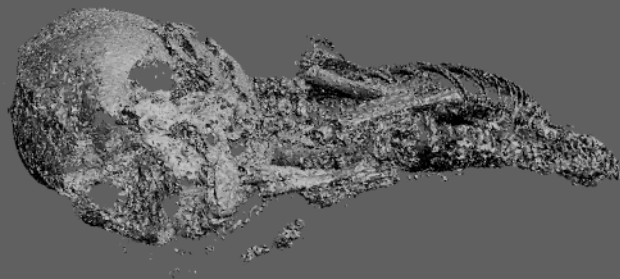

Supplement: Supplementary figure 1 [file rsos171914supp1.pdf]

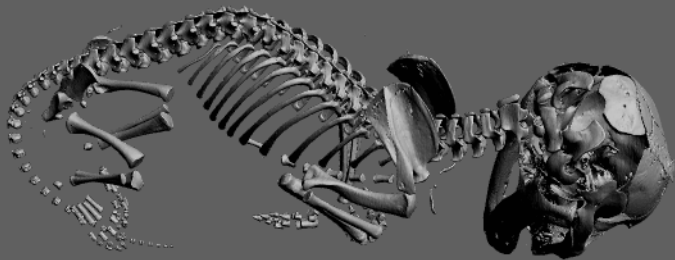

Supplement: Supplementary figure 2 [file rsos171914supp2.pdf]

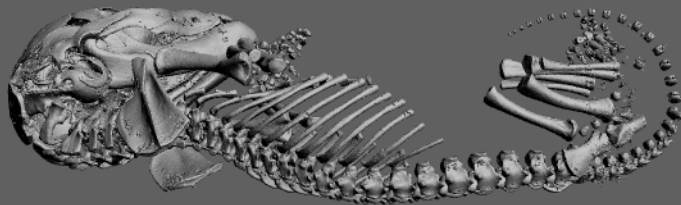

Supplement: Supplementary figure 3 [file rsos171914supp3.pdf]

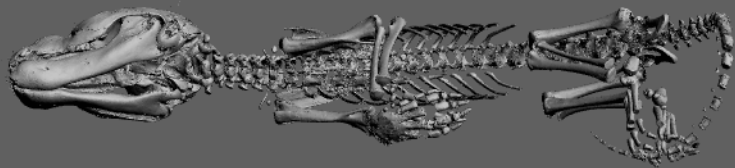

Supplement: Supplementary figure 4 [file rsos171914supp4.pdf]

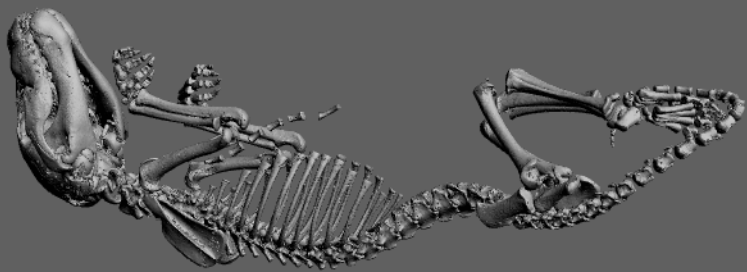

Supplement: Supplementary figure 5 [file rsos171914supp5.pdf]

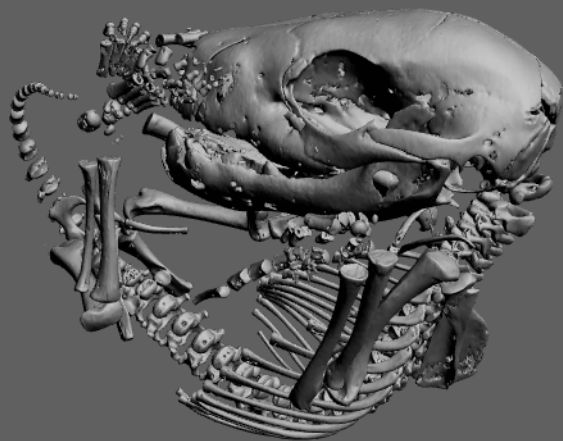

Supplement: Supplementary figure 6 [file rsos171914supp6.pdf]

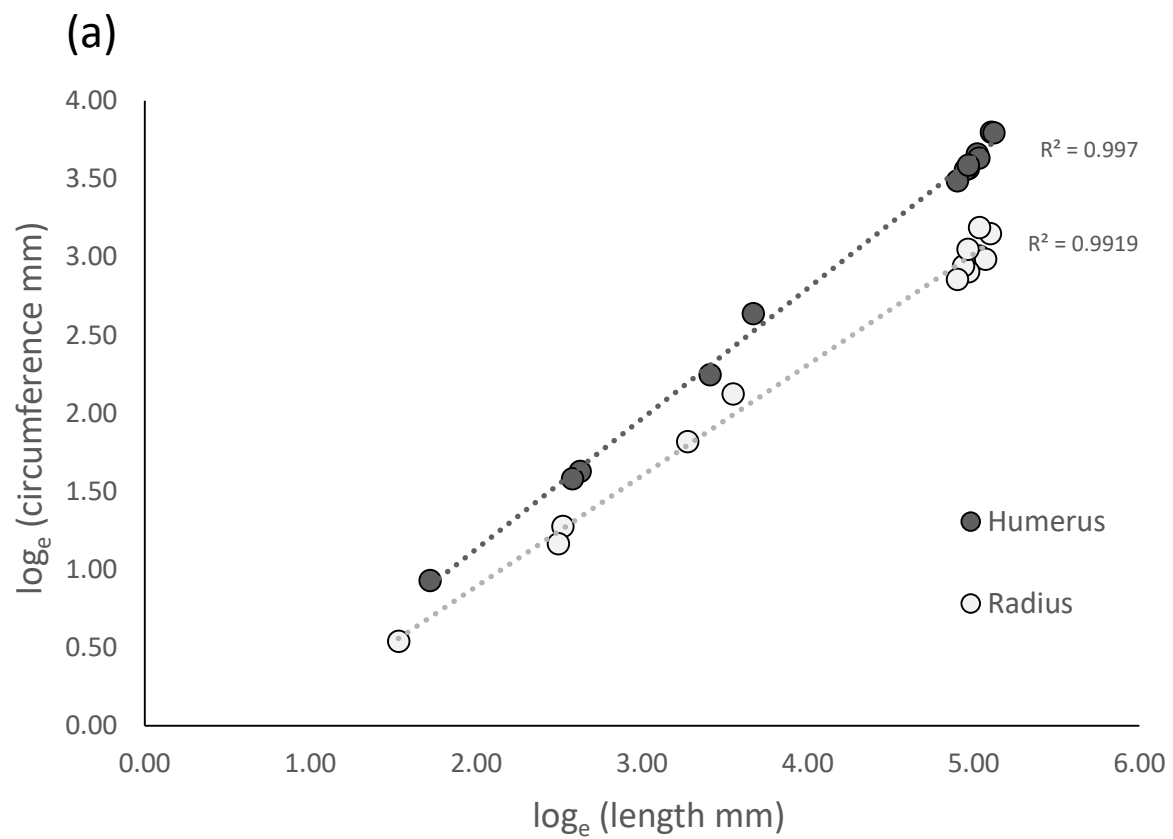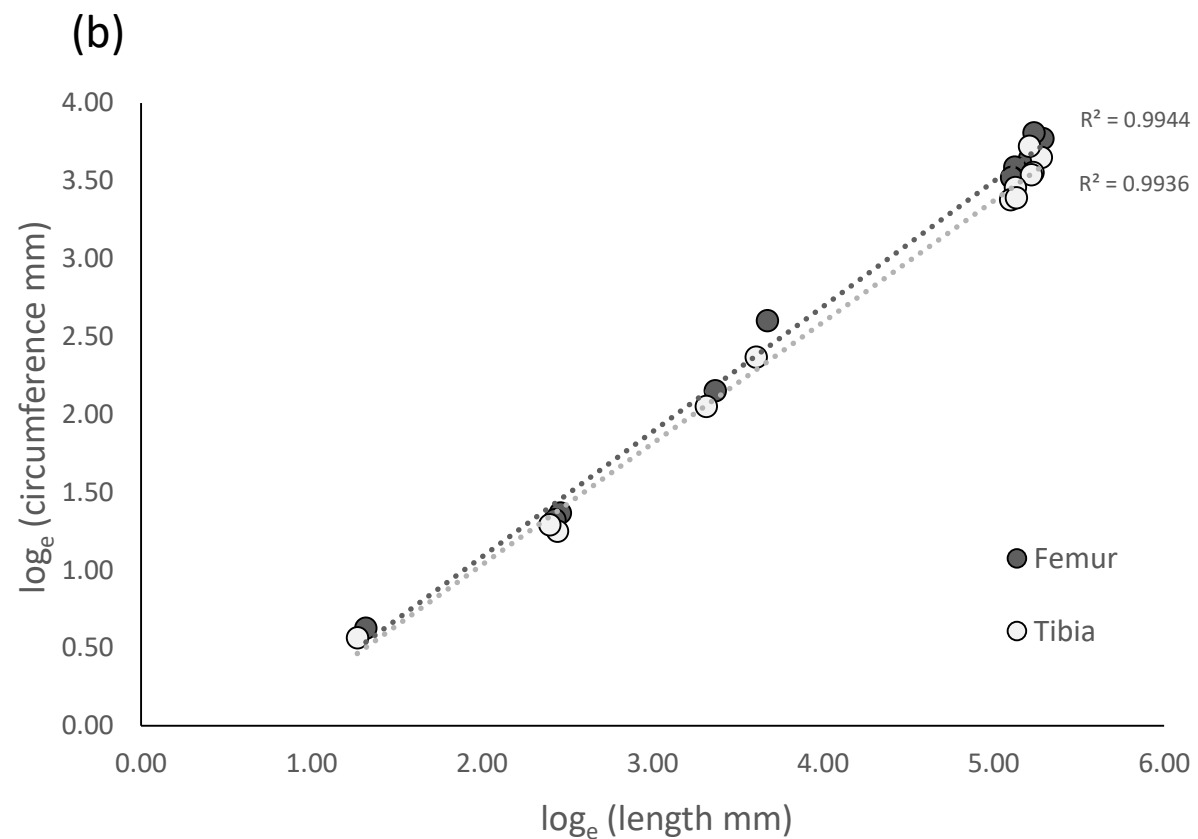

Supplement: Supplementary figure 7 [file rsos171914supp7.pdf]
