# Supplementary material for: Letting the ‘cat’ out of the bag: pouch young development of the extinct Tasmanian tiger revealed by X-ray computed tomography
Source: R Soc Open Sci. 2018 Feb 21;5(2):171914. doi: 10.1098/rsos.171914 (PMC5830782; doi:10.1098/rsos.171914)
Supplement: Supplementary table 1 [file rsos171914supp8.pdf]

	**Raw length data**	**Long bone length (mm)**				**Long bone diameter (mm) - M-L / A-P ave. ****				**Circumference (Pi x diameter) (mm)**			
	**Specimen**	Humerus	Radius	Femur	Tibia	Humerus	Radius	Femur	Tibia	Humerus	Radius	Femur	Tibia
Adult, subadult, juvenile	C5746	165	164	198	196.5	14.19	7.43	13.83	12.24	44.59	23.33	43.44	38.46
	C5749	167.5	153.5	188	183	14.15	7.71	14.36	13.16	44.45	24.23	45.10	41.35
	C5752	151.5	153.25	187.5	187	12.37	6.43	12.35	11.13	38.85	20.21	38.79	34.96
	C5742	153	159.5	183	185.25	12.03	6.31	12.15	10.97	37.79	19.81	38.15	34.45
	C5743	143.5	144	168.5	168	11.24	5.82	11.32	9.48	35.31	18.28	35.55	29.77
	C28718	141	139.5	168	168.5	11.19	6.04	11.53	10.09	35.15	18.97	36.21	31.69
	C5748	134.5	134.5	165.5	164	10.41	5.54	10.79	9.34	32.70	17.40	33.89	29.34
	C5750	143.62	143.35	165	169.5	11.51	6.71	10.75	9.45	36.16	21.07	33.78	29.68
Pouch young	**P762 ***	39.24	34.79	39.42	36.89	4.45	2.66	4.30	3.40	13.96	8.36	13.49	10.67
	**A930 ***	30.27	26.43	28.99	27.51	3.01	1.96	2.74	2.47	9.46	6.17	8.60	7.77
	**A931 ***	13.82	12.45	11.71	11.51	1.62	1.14	1.25	1.11	5.10	3.58	3.92	3.49
	**C5755 ***	13.19	12.11	11.34	10.99	1.55	1.02	1.20	1.16	4.85	3.20	3.75	3.64
	**DZCU * ave.**	5.59	4.62	3.74	3.56	0.81	0.55	0.60	0.56	2.53	1.72	1.87	1.76
	**Natural Log transformed**	Humerus	Radius	Femur	Tibia					Humerus	Radius	Femur	Tibia
Adult, subadult, juvenile	C5746	5.11	5.10	5.29	5.28					3.80	3.15	3.77	3.65
	C5749	5.12	5.03	5.24	5.21					3.79	3.19	3.81	3.72
	C5752	5.02	5.03	5.23	5.23					3.66	3.01	3.66	3.55
	C5742	5.03	5.07	5.21	5.22					3.63	2.99	3.64	3.54
	C5743	4.97	4.97	5.13	5.12					3.56	2.91	3.57	3.39
	C28718	4.95	4.94	5.12	5.13					3.56	2.94	3.59	3.46
	C5748	4.90	4.90	5.11	5.10					3.49	2.86	3.52	3.38
	C5750	4.97	4.97	5.11	5.13					3.59	3.05	3.52	3.39
Pouch young	**P762 ***	3.67	3.55	3.67	3.61					2.64	2.12	2.60	2.37
	**A930 ***	3.41	3.27	3.37	3.31					2.25	1.82	2.15	2.05
	**A931 ***	2.63	2.52	2.46	2.44					1.63	1.28	1.37	1.25
	**C5755 ***	2.58	2.49	2.43	2.40					1.58	1.16	1.32	1.29
	**DZCU * ave.**	1.72	1.53	1.32	1.27					0.93	0.54	0.63	0.56

* pouch young

** average of medial to lateral and anterior to posterior length
